# A Ti_3_C_2_T_x_-Based Composite as Separator Coating for Stable Li-S Batteries

**DOI:** 10.3390/nano12213770

**Published:** 2022-10-26

**Authors:** Ruowei Yi, Yinchao Zhao, Chenguang Liu, Yi Sun, Chun Zhao, Yinqing Li, Li Yang, Cezhou Zhao

**Affiliations:** 1 Department of Chemistry, Xi’an Jiaotong-Liverpool University, Suzhou 215123, China; 2 Stephenson Institute for Renewable Energy, Department of Chemistry, University of Liverpool, Liverpool L69 7ZD, UK; 3 Department of Electrical and Electronic Engineering, Xi’an Jiaotong-Liverpool University, Suzhou 215123, China; 4 Department of Electrical Engineering and Electronics, University of Liverpool, Liverpool L69 3GJ, UK; 5 Dongguan Hongde Battery Co., Ltd., Dongguan 523649, China

**Keywords:** MXene, carbon nanosheets, lithium–sulfur batteries, modified separator

## Abstract

The nitrogen-doped MXene carbon nanosheet-nickel (N-M@CNi) powder was successfully prepared by a combined process of electrostatic attraction and annealing strategy, and then applied as the separator coating in lithium–sulfur batteries. The morphology and structure of the N-M@CNi were characterized by transmission electron microscopy (TEM), scanning electron microscopy (SEM), Raman spectrum, X-ray diffraction (XRD), X-ray photoelectron spectroscopy (XPS), and nitrogen adsorption–desorption method. The strong LiPS adsorption ability and high conductivity are associated with the N-doped carbon nanosheet-Ni modified surface. The modified separator offers the cathode of Li–S cell with greater sulfur utilization, better high-rate adaptability, and more stable cycling performance compared with the pristine separator. At 0.2 C the cell with N-M@CNi separator delivers an initial capacity of 1309 mAh g^−1^. More importantly, the N-M@CNi separator is able to handle a cathode with 3.18 mg cm^−2^ sulfur loading, delivering a capacity decay rate of 0.043% with a high capacity retention of 95.8%. Therefore, this work may provide a feasible approach to separator modification materials towards improved Li-S cells with improved stability.

## 1. Introduction

Over the past decades, lithium–sulfur (Li–S) batteries have attracted tremendous attraction as a substitute for lithium–ion batteries due to their superior theoretical capacity (1672 mAh g^−1^) and outstanding specific energy density (2600 Wh kg^−1^). Many advances based on these attractive features continue to play an important role in the future development of Li–S batteries. However, several intrinsic issues have impeded the path towards the practical application of Li–S batteries, for example, (i) the insulating nature of sulfur and sulfides produced during the discharge process [1], (ii) a large volumetric expansion during discharge [2], and (iii) the dissolution of the high-order lithium polysulfides (LiPS) from the cathode to the organic electrolyte resulting in severe loss of active material and the contamination of lithium anode. Among them, the so-called “shuttle effect”, the movement of the LiPS between cathode and anode, consequently results in the capacity fading and Coulombic efficiency degradation [3].

Substantial efforts have been made to tackle these problems, including designing precisely nanostructured sulfur hosts, i.e., graphene-based nanomaterials [4,5], nitrogen-doped nano-structured carbonaceous materials [6,7,8], two-dimensional (2D) titanium carbide (MXene) [9,10] and so on. These host materials usually provide electron conductivity refinement for sulfur or absorption of LiPS, separately or simultaneously, and significant progress on specific capacity and cycling stability has been made through these works. Among all 2D nanomaterials, MXene, especially Ti_3_C_2_T_x_, holds great promise for high-performance stable Li–S batteries [11], mainly due to its high conductivity and strong interaction between polysulfides and MXene functional groups [12]. Works using MXene as sulfur host of Li–S battery have shown attractive improvement, especially in capacity and cycling stability amelioration [13]. Recently, Bao et al. [14] and Wang et al. [15] reported on the N-doped MXene nanosheet as the sulfur host material and proved its ability in regulating the LiPS. This MXene-doping strategy has further broadened its application in Li–S batteries.

Apart from the strategies involving host materials, another attractive approach to tackle these problems is to insert an extra layer between the cathode and the separator [16,17]. This layer is presented as a form of free-standing interlayer [18,19] or more commonly, as a coating on the separator towards the cathode [20,21,22,23,24,25]. In general, this cathodic interlayer serves several functions in the Li–S cell by (i) shielding the LiPS diffusion to prevent the shuttle effect [16], (ii) acting as an upper current collector to facilitate the redox kinetic, and (iii) reactivating the “dead sulfur species” [26] and increasing sulfur utilization. To facilitate these demands, the coating should be composed of materials with conductive and LiPS-attractive properties. Efforts have been made to use conductive carbonaceous materials such as carbonized paper [27,28], graphene [29], multi-walled carbon nanotube [30], and carbon black [31,32], etc., as an interlayer to accelerate the redox process, resulting in improved capacity performance. Nevertheless, these strategies are usually hard to satisfy the long-term cycling stability due to the lack of chemical absorption for LiPS. Bearing this concern in mind, many polar transition metal oxides/carbides/sulfides, such as TiO_2_ [33], Fe_3_C [34], and Co_9_S_8_ [35], have been used as the interlayer to enhance the LiPS absorption. However, most of these materials have lower electrical conductivity than common conductors, which would increase the internal impedance and deteriorate the re-utilization of the LiPS. Therefore, it is important to modify the separator using novel materials with good conductivity and the ability to attract LiPS at the same time.

MXene, standing out for its high conductivity and strong LiPS interaction, has proved itself in improving the Li–S battery performance as a MXene film coating on the commercial Celgard separator [36]. Although many modified separators using MXene have achieved considerable improvement in specific capacity performance, they are usually applied to cathode with an S-loading of 1–2 mg cm^−2^ [20,21,37,38], resulting in a low energy density. Hence, the challenge is to develop a coating material with both highly conductive and LiPS interactive features and to be able to promote the performance of high S loading cathode.

Herein, we designed and fabricated a nitrogen-doped MXene@nitrogen-doped carbon nanosheet-nickel (N-M@CNi) composite as the separator coating material. The Ni, N-doped carbon nanosheets were introduced by simply annealing the mixture of dicyandiamide, Ni salt, glucose, and MXene. During the annealing, the dicyandiamide absorbed on the MXene surface through electrostatic attraction performed as the nitrogen source of the N-doping [14], producing the N-doped MXene. Such N-doped MXene has been reported to possess more LiPS adsorption sites than those of the pristine one [23]. On the other hand, the N-doping of carbon nanosheets can increase the specific area of the composite, which also provides better LiPS absorption and conductivity than the pristine sp^2^ bonding carbon structure [6,39,40]. Interestingly, some nano-scaled transitional metal particles, such as Co [41,42,43], Fe [44], and Ni [44,45,46] from the VII B group, have also showed a positive effect in LiPS absorption and conversion. Moreover, the LiPS redox can be further enhanced by the comprehensive effect of the N-doped carbon-coated Co or Co_3_O_4_ nano-particles [41,47].

Inspired by these results, we innovatively incorporated the nano-scaled Ni and the N-doped carbon with the MXene to build an interlayer material that is expected to integrate all the merits and present a synergistic effect. Indeed, the separator decorated by the N-M@CNi showed outstanding characteristics on the Li–S batteries, including a high reversible capacity (~1300 mAh g^−1^ at 0.2 C) and an encouraging capacity from a high sulfur loading cathode of 3.18 mg cm^−2^ (662 mAh g^−1^ at 0.2 C after 100 cycles). These favorable results exhibit comprehensively superior performance than those of previous reported similar materials, indicating that the surface modification of the N-doped MXene by the Ni, N-doped carbon nanosheets offers a novel strategy for benefiting the electrochemical performance of Li–S batteries.

## 2. Materials and Methods

### 2.1. Preparation of the Delaminated Ti_3_C_2_T_x_ (MXene)

To prepare the etched Ti_3_C_2_T_x_, 2 g of lithium fluoride (LiF, Aladdin, Shanghai, China, 99.9% metals basis) was added to 40 mL 9 M hydrochloric acid in a Teflon beaker, followed by a 400 rpm stirring for 30 min. Then, 2 g of Ti_3_AlC_2_ powder (MAX, 200 mesh, 11 Technology Co., Ltd., Jilin, China) was carefully added to the above acid mixture. The etching was conducted at 35 °C with 24 h continuous stirring. The resulting suspension was centrifugally separated (3500 rpm, 10 min), then the supernatant was carefully removed. The sediment was added with a total 160 mL deionized water (DI water) and shaken with hand to obtain a homogeneous suspension. Afterward, the suspension was ultrasonicated (750 W, 10 min) and again centrifugally separated (3500 rpm, 10 min). These steps were repeated until the pH value of the supernatant reached 5.

To delaminate the etched Ti_3_C_2_T_x_, 160 mL ethanol was added to the obtained sediment and centrifugally separated at 10,000 rpm for 10 min. The resulting sediment was collected and added with 20 mL DI water and then shaken by hand. The suspension was ultrasonicated (750 W, 20 min) and repeatedly centrifugally separated (350 rpm, 3 min) to collect the dark green supernatant. The resulting suspension was delaminated Ti_3_C_2_T_x_ suspension (MXene suspension). By filtrating a certain volume of MXene suspension and weighing the dried MXene film, the concentration of the resulting MXene suspension was determined as 10 mg mL^−1^.

### 2.2. Preparation of the Nitrogen-Doped MXene@nitrogen-Doped Carbon Nanosheet-Nickel (N-M@CNi) Composite

A total of 75 mg of the nickel acetate tetrahydrate (Ni(Ac)_2_•4H_2_O, Aladdin, 99.9% metals basis) was added to 30 mL of the prepared MXene suspension (10 mg mL^−1^). After 10 min stirring to dissolve the Ni(Ac)_2_•4H_2_O, 1500 mg of dicyandiamide (DCDA, Aladdin, 99%) was added to the suspension, followed by the addition of 150 mg glucose (AR). After adding 20 mL of DI water, the mixture was stirred for 20 min to ensure homogeneity. The mixture was vacuum dried at 60 °C until all solvent was evaporated. The dark remnant was collected after being cooled to room temperature at vacuum state, and then hand-grounded and placed in a corundum crucible. Then, it was heated at 600 °C for 2 h, followed by 800 °C for 2 h, in an argon flow. All the ramping rates were 5 °C min^−1^. The resulting black powder product was named as N-M@CNi. By comparing the mass difference between the final product (488.4 mg) and the starting MXene (300 mg), the content of the nitrogen-doped carbon nanosheet-nickel component was estimated as 38.6 wt.%. For comparison, the composite without Ni content was prepared by washing the prepared N-M@CNi with excessive HCl solution, and subsequently centrifugally washed with DI water and vacuum dried. The resulting product was named N-M@C. By comparing the weight of solid before and after acid-etching, the Ni content in N-M@CNi composite was estimated as 8.6 wt.%.

### 2.3. Preparation of the N-M@CNi Modified Separator

In a typical process, 120 mg N-M@CNi, 120 mg super-P, and 24 mg poly (vinylidene fluoride) (PVDF) were mixed and grounded, followed by the addition of 2.8 g N-methyl-2-pyrrolidinone (NMP, Aladdin, Shanghai, China, AR, >99%). The slurry was magnetically stirred for 12 h and coated on Celgard 2325 separator by the blade coating method. The modified separator was vacuum dried at 60 °C for 12 h, with an areal coating density of ~0.7 mg cm^−2^. The N-M@C modified separator preparation followed the same process, except that the N-M@CNi was replaced with the N-M@C. The carbon black (CB) modified separator was prepared via the same process, except for the addition of N-M@CNi.

### 2.4. Physical Characterization

The electroconductivity of different separators was measured using the four-probe test method. The specific surface area and the pore size distribution measurement were performed on a Beishide (Beijing, China) 3H-2000PS2 instrument, and the analysis was conducted via the Brunauere–Emmette–Teller (BET) method. The micromorphology was observed by the scanning electron microscope (SEM, Hitachi S4700, Tokyo, Japan) and the transmission electron microscopy (TEM, FEI Talos F200×, Thermo Fisher Scientific Inc., Waltham, Massachusetts, USA). The element distribution of the sample was characterized using the energy-dispersive X-ray spectroscopy (EDS). The crystal structure was investigated by an X-ray diffractometer (XRD, D8 Advance, Bruker, Karlsruhe, Germany) using a Cu Kα radiation source from 3° to 80°, along with a Raman spectrometer (Jobin YvonXploRA, HORIBA Scientific, Kyoto, Japan).

### 2.5. Electrochemical Characterization

Electrode preparation: The S/C electrode was fabricated by mixing 60 wt.% sulfur with 30 wt.% super-P as a conducting agent and 10 wt.% PVDF as a binder in the NMP. After being magnetically stirred for 12 h, the slurry was coated on an aluminum foil current collector by a blade-coating machine, followed by vacuum desiccation at 60 °C for 12 h. The prepared electrodes were cut into discs of diameter 13 mm for coin cell assembling. The mass loading of the sulfur was 1.0–3.18 mg cm^−2^, tailored by the thickness of the slurry coating. The typical sulfur loading was ~1.5 mg cm^−2^ if not specifically denoted.

Coin cell preparation: The prepared modified separator was cut into discs of diameter 18 mm, and placed with the coating facing towards the S electrode. The electrolyte was 1.0 M lithium bis (trifluoromethane sulfonyl) imide (LiTFSI) in dioxolane (DOL) and dimethoxyethane (DME) (1:1 by volume) with a 1.0 wt.% LiNO_3_ additive. To better evaluate the performance of the interlayer, the electrolyte was overloaded with the electrolyte/sulfur (E/S, μL/mg) ratio of 20:1 [48]. For the cell without an interlayer, an E/S ratio of 10:1 was used.

Symmetric coin cell preparation: To estimate the electrocatalysis effect on the polysulfide, the symmetric coin cell was tested to eliminate the influence of the reaction between the electrolyte and Li anode. A 0.02 M Li_2_S_6_ solution was prepared by adding the stoichiometric amount of Li_2_S and S_8_ in the electrolyte and stirring for 6 h at 40 °C. Two pieces of modified separators with their uncoated-face attached were placed in the coin cell. Sixty μL Li_2_S_6_ solution was applied in each cell. Two bare separators were used in the blank symmetric cell. To measure the influence of the coating on the ionic conductivity of electrolyte, the stainless-steel symmetric coin cells were assembled with different separators. The thickness of Celgard, CB, and N-M@CNi separators were measured to be 16.4, 26.1, and 30.5 μm, respectively. The area of stainless-steel disc was 2.0 cm^−2^.

Cycling test, open circuit potential measurement, and cyclic voltammetry test: The cycling test was conducted between 1.6–2.7 V (vs. Li/Li^+^) using 1672 mAh g^−1^ as the theoretical specific capacity. The cyclic voltammetry (CV) scanning rate was 0.1 mV s^−1^ between 1.5–2.7 V using the Autolab PGSTAT302N electrochemical workstation. For the symmetric cell, the CV was performed at 10 mV s^−1^ in the range of −1 V to 1 V. The electrochemical impedance spectroscopy (EIS) measurements were conducted on the same workstation with an amplitude of 5 mV and a frequency range of 10^−2^–10^5^ Hz.

## 3. Results

**Scheme 1:**
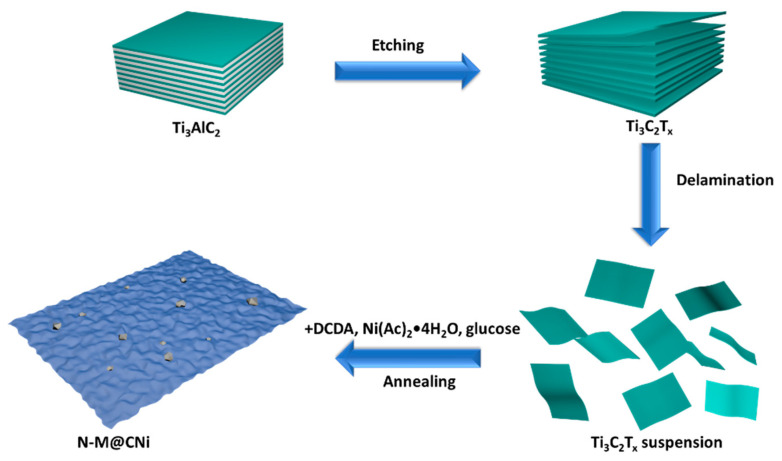
The fabrication process of the N-doped MXene@carbon nanosheets-Ni (N-M@CNi).

Scheme 1 shows the process from the starting material of MAX phase to the last product of N-M@CNi. The aluminum layer was selectively etched from the MAX phase, resulting in a 2D Ti_3_C_2_T_x_ material. After the delamination process, the resulting Ti_3_C_2_T_x_ suspension was mixed with Ni^2+^, DCDA, and glucose successively, and the in situ decoration of N-doped carbon nanosheet with nano-sized Ni particles was formed during the one-step annealing synthesis.

**Figure 1:**
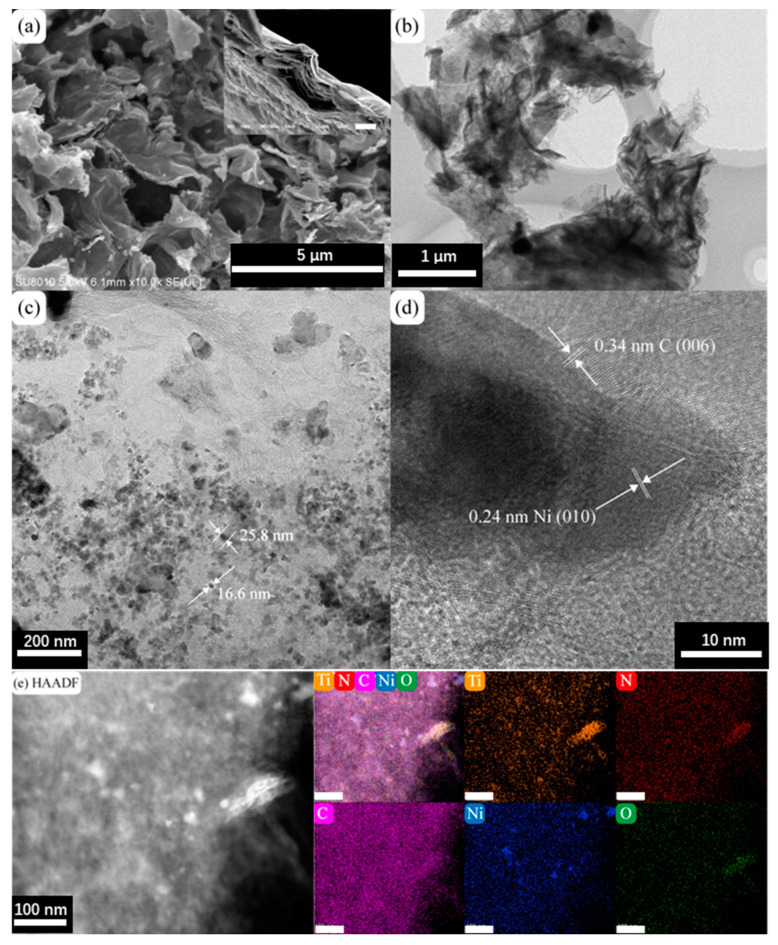
(**a**) SEM image and (**b**) TEM bright-field image of N-M@CNi. The insert is an SEM cross-section of a Ti3C2Tx filtrated film (scale bar, 5 μm). (**c**) The TEM bright-field image of the carbon nanosheets showing the Ni nanoparticles and their typical diameters. (**d**) The HRTEM image of the Ni and C lattice fringes. (**e**) HAADF images and corresponding elemental maps of Ti, N, C, Ni, and O elements. The unlabeled bars in (**e**) represent 100 nm.

The morphology of the prepared N-M@CNi was characterized by SEM and TEM, as shown in Figure 1a,b. The SEM image of N-M@CNi displays that the MXene sheets are coated with crumpled carbon nanosheets, which were formed during the annealing process. Meanwhile, the cross-section in the insert exhibits compactly stacked MXene sheets. This comparison indicates the surface modification has a prominent effect on preventing the self-stacking of MXene. Additionally, the specific surface area and porosity information of the N-M@CNi were measured by the nitrogen adsorption–desorption isotherm method (Figure S1). From the multi-point measurement, the specific surface area of N-M@CNi is 101.8 m^2^ g^−1^, which is larger than the reported value of Ti_3_C_2_T_x_ powder (21.05 m^2^ g^−1^) [49] or film (98 m^2^ g^−1^) [50]. The nitrogen adsorption–desorption isotherm curve in Figure S1a features a prominent H3 hysteresis loop, typical for type-IV curves. The surged adsorption nitrogen near 0.0 P/P_0_ and the hysteresis between 0.5 and 1.0 P/P_0_ are probably associated with the mesopores and macropores in the N-M@CNi [14,23,51]. On the contrary, the un-modified MXene powder (Figure S1b) is found to have a much less specific area (41.4 m^2^ g^−1^) and micropores volume than that of the N-M@CNi. These results suggest that the surface of N-M@CNi has been modified from the carbon nanosheets. A bright-field TEM image of the N-M@CNi (Figure 1c) shows that Ni nanoparticles are randomly distributed on carbon nanosheets, with their diameters ranging from 10 to 50 nm. These nanoparticles are formed during the annealing and wrapped by the carbon structure, as evidenced by the lattice fringes in the high-resolution transmission electron microscopy (HRTEM) (Figure 1d). The high-angle annular dark-field (HADDF) characterization and the EDS mapping were used to characterize the elemental distribution of the N-M@CNi. As exhibited from Figure 1e, the N element distributes evenly on the whole surface of the N-M@CNi, indicating an overall N-doping both in the carbon nanosheets and the Ti_3_C_2_T_x_ sheets is achieved. This is further confirmed by XPS characterization. The clustered Ni nanoparticles in the Ni elemental map correspond to the high contrast points in the HADDF image, which demonstrates that the position of Ni nanoparticles is indeed within the carbon nanosheets.

**Figure 2:**
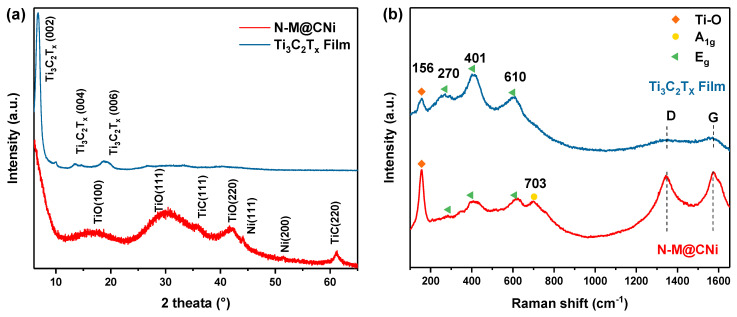
(**a**) XRD patterns and the (**b**) Raman patterns of N-M@CNi powder and Ti_3_C_2_T_x_ film.

Further nanostructure analysis of the synthesized N-M@CNi was carried out by XRD and Raman spectrum, as shown in Figure 2. The XRD pattern (Figure 2a) of the pure Ti_3_C_2_T_x_ film shows only peaks of delaminated Ti_3_C_2_T_x_, including the prominent (002) peak at 6.8°. This is direct evidence of the successful delamination of the Ti_3_C_2_T_x_ sheets [52]. For the pattern of the N-M@CNi sample, the low-angle shifted (002) peak reflects an enlarged interlayer spacing of the Ti_3_C_2_T_x_ sheets [53], indicating that the formation of the nitrogen-doped carbon nanosheets has a strong interaction with the MXene sheets’ surface. The broad peaks at about 17°, 30°, and 43° are assigned to a small amount of nanosized TiO (002), (111), and (220) planes, correspondingly (JCPDS card no. 23–1078). Additionally, two minor peaks at 35° and 61° could be the (111) and (220) planes for the TiC, respectively (JCPDS card no. 32–1383). A small amount of TiO and TiC are formed during the heat process. This is probably due to the conversion of the unstable Ti–O group on the MXene surface and the rearrangement of Ti_3_C_2_T_x_ after the detachment of surface groups, respectively. Two minor peaks at about 44° and 51° represent the (111) and (200) planes of Ni particles [54]. These two samples were further examined by Raman spectroscopy. As shown in Figure 2b, the bands at 156 cm^−1^ (denoted by orange diamonds) are associated with the stretching vibration of the Ti–O bond [55,56]. The enhanced band intensity of the N-M@CNi spectrum may be caused by the partial oxidation during the annealing process. Three bands, denoted by green triangles (270, 401, and 610 cm^−1^), are related to the E_g_ group vibrations, including the in-plane (shear) mode of Ti, C, and surface functional group atoms. The yellow circle marked band at 703 cm^−1^ is indexed to the A_1g_ symmetry out-of-plane vibrations of Ti and C atoms [56,57,58]. At around 1350 cm^−1^ and 1580 cm^−1^, two broad and weak bands (D and G) are identified in the Ti_3_C_2_T_x_ film spectrum, which can be explained by some free carbon and the disorder of the sample [59]. Meanwhile, in the spectrum of N-M@CNi, the D and G become much more prominent, indicating the presence of graphitic carbon nanosheets [56,60]. More details of the deconvoluted result can be found in Figure S2 and Table S1.

**Figure 3:**
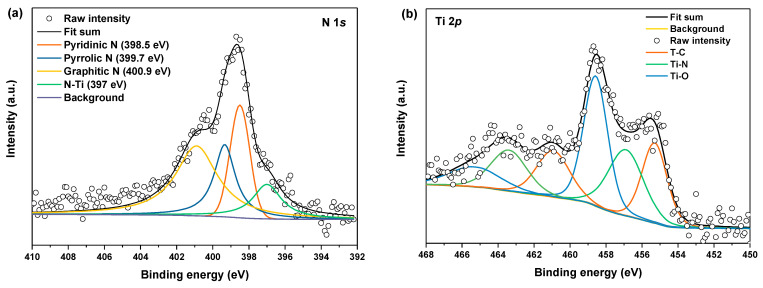
The deconvoluted XPS of (**a**) N 1*s* and (**b**) Ti 2*p* of the N-M@CNi sample.

To further evaluate the N-doping in N-M@CNi, XPS analysis was conducted and the deconvoluted results of N 1*s* and Ti 2*p* spectra are shown in Figure 3a,b, respectively. Deconvoluted peaks at 398.5 eV, 399.7 eV, 400.9 eV, and 397 eV are indexed as pyridinic N, pyrrolic N, graphitic N [61,62], and Ti–N bond [14,63], respectively. The Ti–N bond at 456.9 eV is further confirmed by the Ti 2*p* deconvoluted result (Figure 3b) [23]. The N-doping is achieved both in carbon nanosheet and Ti_3_C_2_T_x_ in the N-M@CNi. The dual doping effect is essential for N-M@CNi to enhance battery performance as a separator coating material, which is supported by previous reports. The N-doping in sp^2^ carbon materials is found not only to facilitate the conductivity [64] but also to absorb LiPS [65] and kinetically accelerate their conversion [6]. Compared to the pristine Ti_3_C_2_T_x_, the N-doped Ti_3_C_2_T_x_ has higher chemical adsorption energy, preventing the diffusion of the dissolved polysulfide and consequently promoting the utilization of active material [14,23].

**Figure 4:**
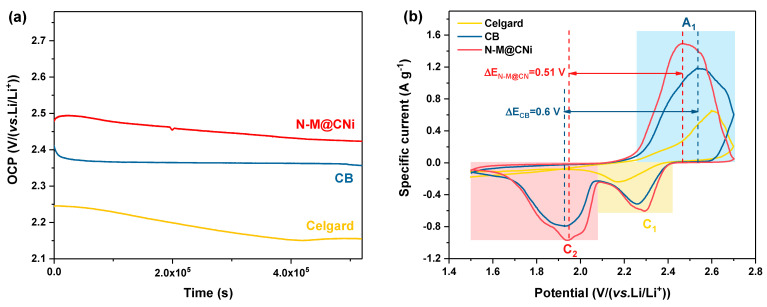
(**a**) OCP test of cells with N-M@CNi or CB-modified separator, or with pristine Celgard separator. (**b**) Cyclic voltammetry curves of cells with N-M@CNi- or CB-modified separator, or with pristine Celgard separator. The sulfur loading in the tested cathode was ~2.3 mg cm^−1^.

For Li–S batteries, one of the major challenges is the self-discharge problem induced by the shuttle effect. It is reported that there are almost 30% of capacity lost via self-discharge within hours [66], which is a severe drawback in practical application. To evaluate the effect of self-discharge on the N-M@CNi-modified separator, an open circuit voltages (OCP) test was conducted on cells with CB and N-M@CNi-modified separator, as well as the pristine cell with Celgard separator (Figure 4a). The cell with a pristine Celgard separator starts with an OCP of 2.25 V and ends at 2.15 V, experiencing the most severe potential variation rate of −6.92 × 10^−1^ mV h^−1^. By comparing with the voltage of the first discharge plateau (~2.3 V) in the following galvanostatic charge/discharge curves, it is clear that a dramatic spontaneous reduction in elemental sulfur and high-order polysulfides occur in the pristine Li–S cell. The cell with the N-M@CNi separator presents the highest initial potential of 2.47 V among three cells and retains 2.42 V after over 5.2 × 10^5^ s (potential variation rate = −2.77 × 10^−1^ mV h^−1^), as shown in Figure 4a. It also has a higher OCP value than those of bi-layer graphene film [67] and graphene oxide [66] interlayers at the same test duration. In contrast, the cell with CB-modified separator shows an initial potential of 2.4 V, and a rapid degrading is observed during the first 0.5 × 10^5^ s, ending in 2.35 V after only 5.2 × 10^5^ s (−2.88 × 10^−1^ mV h^−1^). This prominent anti-self-discharge effect of the N-M@CNi separator is due to its adsorption effect that effectively inhibits the loss of the LiPS.

The electrochemical performance of different separators in Li–S cells was probed by the CV method, as shown in Figure 4b. The CV test was performed at a scan rate of 0.1 mV s^−1^ between 1.5 and 2.8 V. Cells with CB and N-M@CNi separators contain two cathodic peaks (C_1_ and C_2_) and one anodic peak (A_1_). The A_1_ peak corresponds to the oxidation of the Li_2_S_2_/Li_2_S to sulfur, while the C_1_ and C_2_ represent the reduction in the sulfur to high-order LiPS and the formation of Li_2_S_2_/Li_2_S [68]. The pristine cell with Celgard separator shows only one anodic peak and one cathodic peak. The second step reduction in the high sulfur loading electrode is retarded because of the prominent accumulation of the insulating high-order LiPS during the first-step reduction, causing great polarization of the cell. Since the absence of an electronic conductive coating in the Celgard cell, the accumulated insulating polysulfides cannot be timely reduced, resulting in great electrochemical polarization for the second-step reduction. Therefore, the C_2_ peak shifts beyond the voltage window [69,70]. To evaluate the redox kinetics of the cell [69,71,72], we also calculated the potential difference between the anodic peak and the cathodic peak. As shown by the potential difference ΔE between A_1_ and C_2_ in Figure 4b (as denoted by the distance between dash lines), the ΔE_N-M@CNi_ is smaller than ΔE_CB_. Therefore, it is clear that the N-M@CNi cell facilitates a better kinetics than that of the CB cell.

**Figure 5:**
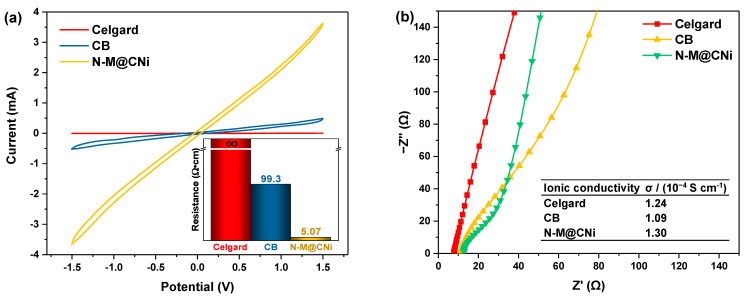
(**a**) The CV of the symmetric cells (coating/Celgard/Celgard/coating) with different separators. The insert shows the resistance of different separators measured by the four-probe method. (**b**) The impedance plots of the stainless steel/modified separator/stainless steel cells with different separators. The inserted table summarizes the calculated ionic conductivity values of the Celgard, the CB, and the N-M@CNi separators.

A CV test of symmetric cells was used to evaluate the redox conversion kinetic of LiPS on the coating material of the separator. At a scanning rate of 10 mV s^−1^, as seen in Figure 5a, it is observed that the N-M@CNi-modified separator has a higher current than the CB ones at the same potential, while the Celgard separator displays almost no current variation during the voltage window. This comparison indicates a very fast electrochemical reaction of the LiPS on the N-M@CNi coating, as a result of the co-operation of the stronger LiPS absorption as well as the low charge transfer resistance (as presented by the insert in Figure 5a, where the electronic resistance of N-M@CNi separator is almost two orders of magnitude lower than that of the CB) [73].

Apart from the electronic conductivity, the ionic conductivity was calculated from the impedance of the cell containing only the modified separator as the electrode (Figure 5b). Unlike the typical plots consisting of a high-frequency semicircle and a low-frequency straight line, the impedance plots of these modified separators show almost only the straight-line part. This indicates that nearly all the charge carriers in these cells are ions [37]. Therefore, the ionic conductivity of the liquid could be calculated via the formula [74]:

σ = *I*/(*R_b_A*) (in S cm^−1^)

where σ is the ionic conductivity, *I* is the thickness of the separator, *R_b_* is the bulk resistance which can be obtained from the intercept of the fitted straight line on the real axis (Z’), and *A* represents the area of the stainless-steel disc since the separator is larger than that of the stainless steel disc. The exact values of parameters for calculation are listed in the Section 2. The ionic conductivity is 1.30 × 10^−4^ S cm^−1^ for the N-M@CNi separator, slightly higher than that of the CB (1.09 × 10^−4^ S cm^−1^) and the Celgard separators (1.24 × 10^−4^ S cm^−1^). These data confirm that the ionic conductivity of the N-M@CNi separator is at the same level as the commercial Celgard ones, which has no negative effect on ionic transportation in the Li–S battery. This feature, along with the better electron conductivity of the N-M@CNi separator, is a crucial basis for the enhanced performance of the N-M@CNi battery at the high current rate, as discussed in the following part.

**Figure 6:**
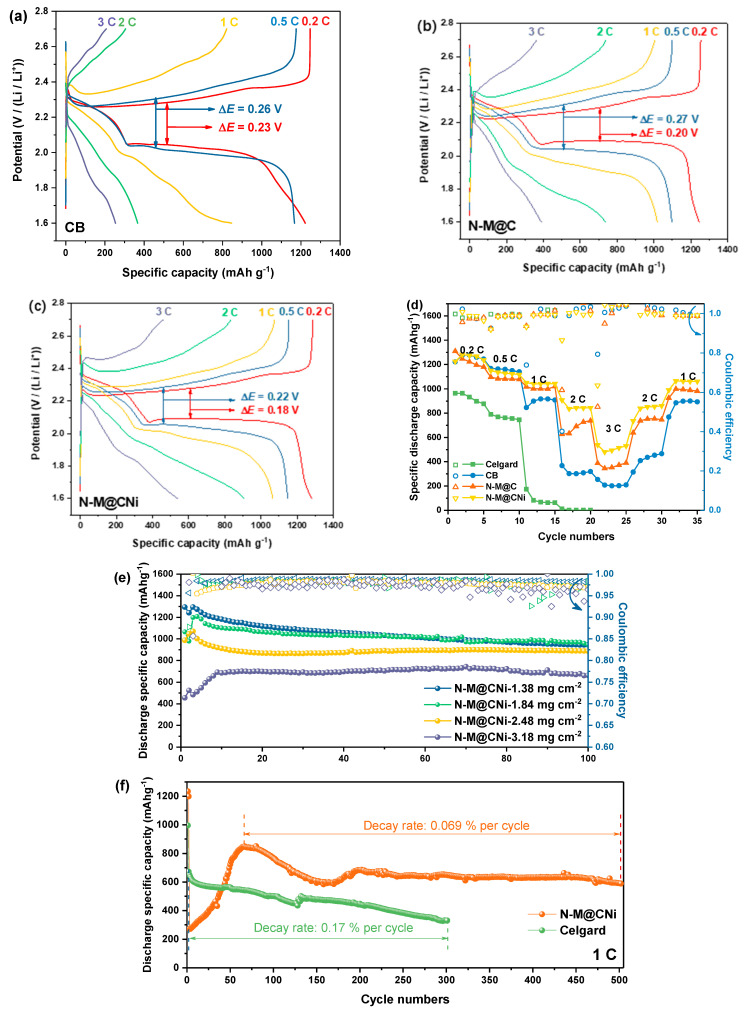
The typical galvanostatic charge and discharge (GCD) curves of (**a**) CB, (**b**) N-M@C, and (**c**) N-M@CNi cells at 0.2 C, 0.5 C, 1 C, 2 C, and 3 C. The rate performance (**d**) of the Celgard, the CB, the N-M@C and the N-M@CNi cells at a current of 0.2 C. (**e**) The cycling performance comparison of N-M@CNi cells with different sulfur mass loading cathodes at 0.2 C. (**f**) The long-term cycling performance of Celgard and N-M@CNi cells at 1 C.

To highlight the improved electrochemical performance of the N-M@CNi separator, galvanostatic discharge/charge profiles of cells with CB, N-M@C, and N-M@CNi separator are compared and evaluated at different current rates (Figure 6a–c), and their rate performances are shown in Figure 6d. The pristine Celgard cell only delivers capacities of 964 mAh g^−1^ and 790 mAh g^−1^ at 0.2 C and 0.5 C and dived to 174 mAh g^−1^ at 1 C, respectively. At 2 C rate, it provides little capacity and eventually fails at a higher rate. For cells with modified separators, the CB cell shows similar specific capacity values with the N-M@CNi and N-M@C ones at low current rates (0.2 C and 0.5 C). The cycling performance of these cells at 0.2 C also exhibits the same tendency (Figure S3). Nevertheless, the Coulombic efficiency tendency during these 100 cycles indicates that N-M@CNi and N-M@C have a stronger ability to inhibit the shuttle effect than the pure CB. Typical galvanostatic profiles of various cells are shown in Figure 6a–c. As the current density increases, all cells exhibit expanded potential hysteresis and shrunk discharge/charge plateaus, which can be ascribed to the hindered ion at a high current density that results in greater polarization and lower sulfur utilization. The N-M@CNi separator has smaller potential hysteresis (represented by ΔE in Figure 6a,b, the overpotential between the oxidation and the lower reduction plateaus) than the CB and N-M@C ones [51]. When the current is boosted to higher rates (i.e., 1 C, 2 C, and 3 C), the cell using Celgard fails at 2 C and cannot be resumed afterward, while the cell with CB separator delivers much lower capacity than the N-M@CNi ones (846 mAh g^−1^, 367 mAh g^−1^, and 253 mAh g^−1^, compared with 1051 mAh g^−1^, 907 mAh g^−1^, and 538 mAh g^−1^, respectively). The N-M@C cell shows a more pronounced lower capacity than that of the N-M@CNi cell at higher current density (2 C and 3 C), suggesting that the Ni nano-particle plays a crucial role in boosting the redox at higher current density (Figure 6d). Furthermore, the potential hysteresis (ΔE) of the CB and N-M@C cell increases at these high currents, along with the diminishing of discharge/charge plateaus (Figure 6a,b). Meanwhile, the discharge/charge plateaus in the N-M@CNi cell remain distinguishable at the same current rates. This result is in good agreement with the rate test data. Therefore, it can be concluded that the N-M@CNi separator is superior to the CB and N-M@C separator in electrochemistry kinetics, facilitating the redox process within a wide range of current rates.

Whilst the CB cell displays similar specific discharge capacity with that of the N-M@CNi cell at 0.2 C, the cycling stability of these two is observed a noticeable distinction at 0.2 C, as plotted in Figure S4. Both N-M@CNi, N-M@C, and CB cells start from ~1300 mAh g^−1^ and are stabilized at ~940 mAh g^−1^ after 100 cycles, during which period the pristine Celgard cell drops from 900 to 640 mAh g^−1^. The main difference lies in the coating used here, showing the effective promotion of sulfur utilization and cycling stabilization. Despite similar cycling capacity performance, the Coulombic efficiency of the CB cell gradually deviates from the N-M@CNi ones as the cycle continues. Unlike the N-M@CNi cell having a steady efficiency of 98~99% throughout the whole 100 cycles, the Celgard cell, and the CB cell suffer from a continuing degradation, dropping from the initial ~98% to ~90% and ~96%, respectively. Another Coulombic efficiency comparison between N-M@CNi (98.8 ± 0.3%) and N-M@C (98.47 ± 0.5%) cells indicates the incorporation of Ni nanoparticles can not only enhance the rate performance but also improve the reversibility. This difference in the Coulombic efficiency provides intuitive proof that the N-M@CNi has a fairly strong ability to prohibit the shuttle effect, which was the direct rationale for the Coulombic efficiency preservation [26].

To check its polysulfides-inhibiting ability on the cathode with different sulfur mass loading, the N-M@CNi separator was applied to cathodes with areal sulfur loading ranging from 1.38 to 3.18 mg cm^−2^, and the corresponding cycling performance at 0.2 C is shown in Figure 6e. For cathodes with 1.38 and 1.84 mg cm^−2^ sulfur loading, similar cycling performance is observed for 100 cycles. Meanwhile, the 2.48 mg cm^−2^ cathode delivers a maximum capacity of 1070 mAh g^−1^, which is lower than those of the former two cathodes. However, good capacity retention of 888 mAh g^−1^ after 100 cycles is achieved, merely ~55 mAh g^−1^ lower than those of lower sulfur loading electrodes. This performance on sulfur loading adaptability is superior to the previously reported pure MXene-modified separator [36]. When the areal density of sulfur surged to 3.18 mg cm^−2^, the maximum capacity drops to 691 mAh g^−1^. Nevertheless, its capacity remains fairly steady and the value is kept at 662 mAh g^−1^ after 100 cycles, giving a capacity decay rate of 0.043% per cycle with high retention of 95.8%. For the long-term high-rate cycling performance, the N-M@CNi separator also provides considerable improvement for the S/C electrode (Figure 6f). The ascending capacity performance of the first ~60 cycles might be ascribed to the period of full activation process (meaning the LiPS continuous dissolution and adsorption at a high current rate [75]). After that, the N-M@CNi cell exhibits a peak capacity of 848 mAh g^−1^ and retains 588 mAh g^−1^ after 500 cycles. This gives a capacity decay rate of only 0.069% per cycle. The cycling performance at 1 C is better than the reported pure Ti_3_C_2_T_x_-modified separator [36] and many other separators modified with other composites [70,76,77,78,79]. More detailed comparisons between the cycling performance of previous reported MXene- or N-doped carbon-based separator coatings are presented in Table S2. It is noted that the comprehensive cycling performance of the N-M@CNi coating stands out among the similar reported coating materials, even coupled with an unmodified S cathode. During this time the pristine Celgard cell drops from 672 to 330 mAh g^−1^ after only 300 cycles, showing a 0.17% decay rate per cycle. This capacity performance is better than that of the Celgard cell in the rate test (174 mAh g^−1^ at 1 C, in Figure 6d). This can probably be ascribed to the unlimited shuttle effect that severely corroded the Li anode during the previous cycles at lower current densities in the rate test. Meanwhile, the Celgard cell without deep pre-cycles in the long-term 1 C cycling exhibits better capacity performance due to the less-corroded Li anode. Therefore, using the N-M@CNi separator offers better long-term stability with strong LiPS absorption and enhances sulfur utilization at a high rate. On the other hand, the performance of the N-M@CNi separator on cathodes with different mass loading confirms its good capability of different sulfur loading adaption. It is expected that the N-M@CNi separator might expand its potential on the cathode with more advanced sulfur-containing materials for higher demands of sulfur mass loading, facilitating the practical application of the Li–S batteries.

**Figure 7:**
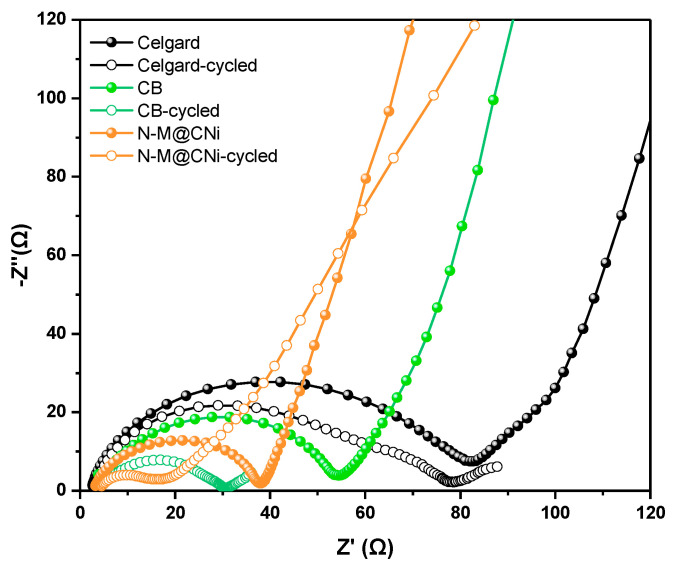
The EIS plots of Celgard, CB, and N-M@CNi cells before (solid dots) and after 10 cycles (hollow dots) at 0.2 C.

The electrochemical impedance spectroscopy (EIS) of Celgard, CB, and N-M@CNi cells was conducted before and after the cycling test, to gain better insight into the N-M@CNi separator. All curves are composed of a semi-circle at high frequency and a subsequent oblique line at low frequency. The relevant Nyquist plot is shown in Figure 7. The diameter of semi-circle representing the charge transfer resistance (R_ct_) [33] follows the sequence of Celgard > CB > N-M@CNi. This impedance ranking is a direct result of the higher conductivity of the N-M@CNi than the other two separators, which is beneficial to optimizing the localized charge transfer of sulfur species [80]. After a ten-time cycle, cells with the interlayer display a shrunken semicircle, indicating the cathode impedance is ameliorated. This is explained by the diffused sulfur species from cathode to the interlayer and is rearranged at more electrochemical facilitated sites, thus the charge transfer of the active materials is reduced [34,81]. However, for the Celgard one, no obvious change is observed from R_ct_. An extra medium-frequency semi-circle appears after the cycles, probably due to the precipitation of the Li_2_S_2_/Li_2_S layer on the cathode surface in the absence of a conductive interlayer [82]. It is worth noting that after cycling the N-M@CNi cell presents much lower impedance than that of the CB cell, demonstrating its superior polysulfide-adsorption performance than the later ones. Further characterization of the cycled N-M@CNi can be found in the deconvoluted XPS results (Figure S4), in which the S 2*p* and Ti 2*p* spectra illustrate the presence of the sulfur species and the Ti-S bond. The polythionate and the thiosulfate peaks in Figure S4a are caused by the residual LiTFSI in the electrolyte and the surface redox between N-M@CNi and the polysulfides [23]. The Ti-S bond indicates the chemical adsorption by the N-M@CNi. All these results confirm an occurrence of the LiPS adsorption during the cycling process.

## 4. Conclusions

In summary, the N-doped Ti_3_C_2_T_x_@carbon nanosheets-Ni composite was synthesized as a separator coating material for improved capacity and cycling stability in Li–S cells. The modified surface and the N-doped structure provide strong LiPS adsorption ability and high conductivity. Using the N-M@CNi separator in the cathode of Li–S cell offers greater sulfur utilization, better high-rate adaptability, and more stable cycling performance compared to the Celgard and CB cells. At 0.2 C the cell with N-M@CNi separator delivers an initial capacity of 1294 mAh g^−1^ and 943 mAh g^−1^ after 100 cycles. When the current increases to 1 C, a steady capacity of 588 mAh g^−1^ is achieved after 500 cycles with a low-capacity decay rate of 0.069% per cycle. This work may provide a feasible approach of separator modification materials toward improved stable Li–S cells.

## Data Availability

The data presented in this study are available on request from the corresponding author.

## Supplementary Materials

The following supporting information can be downloaded at: https://www.mdpi.com/article/10.3390/nano12213770/s1, Figure S1: nitrogen adsorption–desorption isotherm of (a) N-M@CNi and (b) MXene powder. The insert shows the pore size distribution acquired using the Barrett—Joyner–Halenda (BJH) method, Figure S2: the fitting curves of the Raman spectrum of the carbon nanosheet part, Figure S3: the cycling performance at 0.2 C current density of N-M@CN, N-M@C, CB, and Celgard cells, Figure S4: the deconvoluted XPS spectra of the cycled N-M@CN separator: (a) S 2*p* and (b) Ti 2*p.* The adsorption of LiPS and the presence of the Ti-S bond are shown in the deconvoluted results, Table S1: the parameters of the fitting results from the fitting curves in Figure S2, Table S2: The performance comparison of Li–S batteries using MXene and/or N-doped carbon-based cathodic interlayer/separator coating from the literature.
