# Supplementary material for: A Ti3C2Tx-Based Composite as Separator Coating for Stable Li-S Batteries
Source: Nanomaterials (Basel). 2022 Oct 26;12(21):3770. doi: 10.3390/nano12213770 (PMC9658629; doi:10.3390/nano12213770)
Supplement: Supplementary file 1 [file nanomaterials-12-03770-s001.zip › nanomaterials-1929341-supplementary.pdf]

Supplementary Materials

A $Ti_3C_2T_x$-Based Composite as Separator Coating for Stable Li-S Batteries

Ruowei Yi [1,2], Yinchao Zhao [3,4], Chenguang Liu [3,4], Yi Sun [3,4], Chun Zhao[3], Yinqing Li [5], Li Yang [1,*], Cezhou Zhao [3,*]

1 Department of Chemistry, Xi'an Jiaotong-Liverpool University, Suzhou 215123, China
2 Stephenson Institute for Renewable Energy, Department of Chemistry, University of Liverpool, Liverpool L69 7ZD, UK
3 Department of Electrical and Electronic Engineering, Xi'an Jiaotong-Liverpool University, Suzhou 215123, China
4 Department of Electrical Engineering and Electronics, University of Liverpool, Liverpool L69 3GJ, UK
5 Dongguan Hongde Battery Co., Ltd., Dongguan 523649, China
* Correspondence: li.yang@xjtlu.edu.cn (L.Y.); cezhou.zhao@xjtlu.edu.cn (C.Z.)

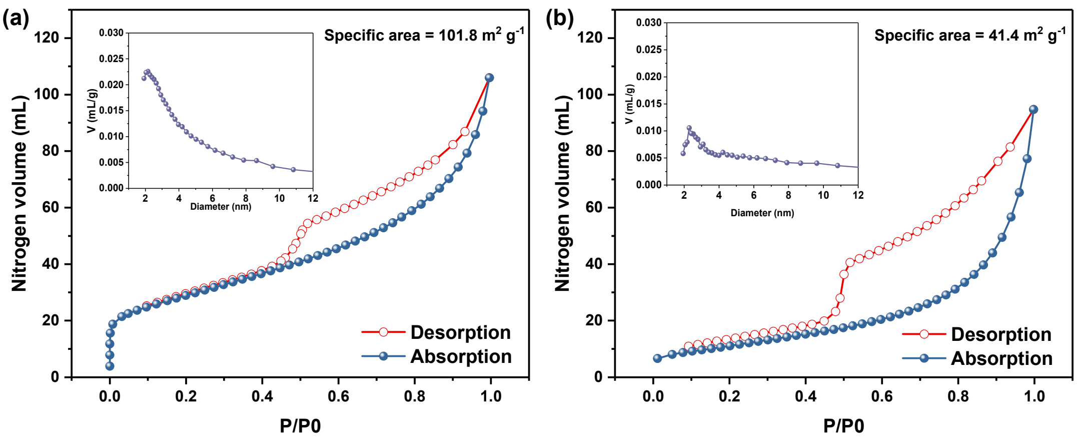

Figure S1. Nitrogen adsorption-desorption isotherm of (**a**) N-M@CNi and (**b**) MXene powder. The insert shows the pore size distribution acquired using the Barrett-Joyner-Halenda (BJH) method.

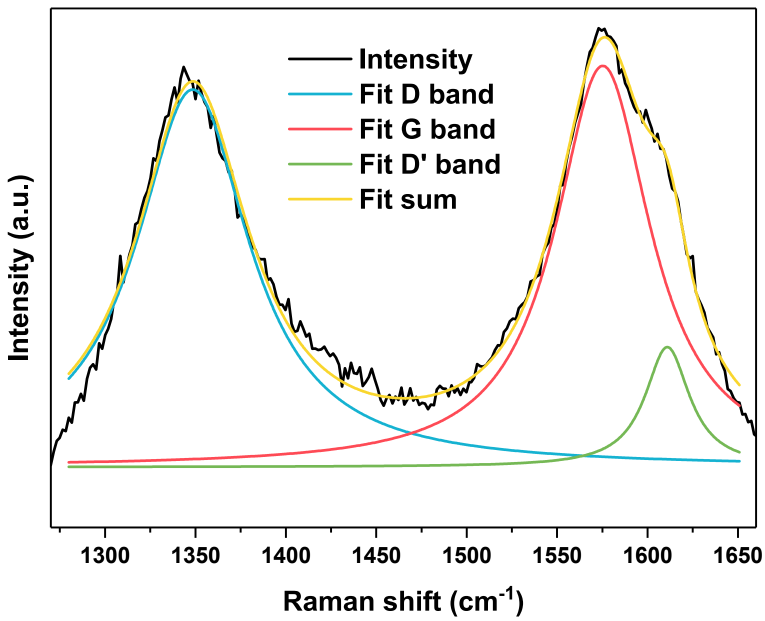

Figure S2. The fitting curves of the Raman spectrum of the carbon nanosheet part.

Table S1. The parameters of the fitting results from the fitting curves in Figure S2.

Bands	D band	G band	D′ band	R-Square (COD)	I_D/I_G
Center (cm^{-1})	1348.4	1575.2	1610.9	0.98	1.1
FWHM (cm^{-1})	75.8	64.2	29.9		
Integrated area	310876.6	280035.7	39043.1		

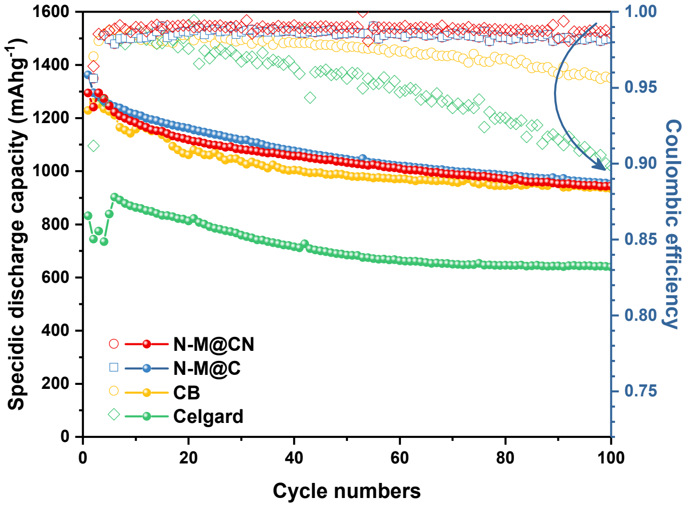

Figure S3. The cycling performance at 0.2 C current density of N-M@CNi, N-M@C, CB, and Celgard cells.

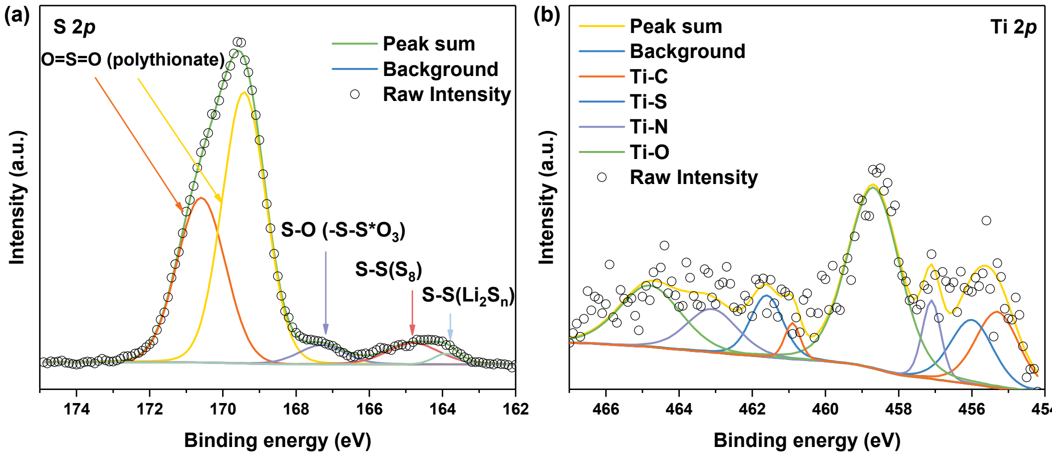

Figure S4. The deconvoluted XPS spectra of the cycled N-M@CNi separator: (**a**) S 2*p* and (**b**) Ti 2*p*. The adsorption of LiPS and the presence of the Ti-S bond are shown in the deconvoluted results.

Table S2. The performance comparison of Li-S batteries using MXene and/or N-doped carbon-based cathodic interlayer/separator coating from literatures.

Composites	Sulfur area loading (mg cm^{-2}) and sulfur specie of the cathode	Specific capacity after cycling (mAh g^{-1})	Current rate and cycle times	Ref.
$Ti_3C_2T_x$	1.2, S/CB	495	1 C, 500 cycles	[36]
Ti_3C_2	0.7–1, S/Ti_3C_2	~400<br>~360	0.5 C, 200 cycles<br>2 C, 200 cycles	[77]
$Ti_3C_2T_x$@Nafion	2, S/CB	415	1 C, 1000 cycles	[20]
N-dope carbon nanofibers/SiO_2/TiO_2	1.8, S/Ketjen black	435	1 C, 500 cycles	[78]
N, P-dual-doped carbon	1.6–2.0, S/acetylene black	440	1 C, 900 cycles	[70]
F, N-co-doped carbon	1.2, S/super-P	640	0.2 C, 500 cycles	[79]
N-M@CNi	1.5, S<br>3.18, S	588<br>662	1 C, 500 cycles<br>0.2 C, 100 cycles	This work
